# Distinct microbial communities degrade cellulose diacetate bioplastics in the coastal ocean

**DOI:** 10.1128/aem.01651-23

**Published:** 2023-12-06

**Authors:** Yanchen Sun, Michael G. Mazzotta, Carolyn A. Miller, Amy Apprill, Mounir Izallalen, Sharmistha Mazumder, Steven T. Perri, Brian Edwards, Christopher M. Reddy, Collin P. Ward

**Affiliations:** 1 Department of Marine Chemistry and Geochemistry, Woods Hole Oceanographic Institution, Woods Hole, Massachusetts, USA; 2 Eastman Chemical Company, Kingsport, Tennessee, USA; Georgia Institute of Technology, Atlanta, Georgia, USA

**Keywords:** cellulose diacetate, plastic pollution, bioplastics, biodegradation, microbial communities, 16S rRNA gene amplicon sequencing

## Abstract

Cellulose diacetate (CDA) is a bio-based plastic widely used in consumer products. CDA is a promising alternative to conventional thermoplastics due to its susceptibility to biodegradation in various environments. Despite widespread evidence for the degradation of CDA, relatively little is known about the microorganisms that drive degradation, particularly in the ocean. Recently, we documented the biodegradation of CDA-based materials (i.e., fabric, film, and foam) in a continuous-flow natural seawater mesocosm on the timescales of months, as indicated by mass loss, enzyme activity, and respiration to carbon dioxide. These findings paved the way for the present study aimed at identifying key microbial taxa implicated in CDA degradation. Analysis based on 16S rRNA gene amplicon sequencing of bacteria and archaea revealed that material type, incubation time, material morphology (e.g., fabric vs film), and plasticizer content significantly influenced the microbial community structure. Differential abundance analysis revealed that bacterial taxa affiliated with the families of *Arenicellaceae*, *Cellvibrionaceae*, *Methyloligellaceae*, *Micavibrionaceae*, *Puniceicoccaceae*, *Spirosomaceae*, and *Thermoanaerobaculaceae*, and the order of *Pseudomonadales* potentially initiated the degradation (i.e., deacetylation) of CDA fabric and film. These taxa were notably distinct from CDA-degrading microbes reported in non-seawater environments. Collectively, the findings lend further support for CDA as a promising next-generation, high-utility, and low-environmental persistence bioplastic material.

**IMPORTANCE:**

Cellulose diacetate (CDA) is a promising alternative to conventional plastics due to its versatility in manufacturing and low environmental persistence. Previously, our group demonstrated that CDA is susceptible to biodegradation in the ocean on timescales of months. In this study, we report the composition of microorganisms driving CDA degradation in the coastal ocean. We found that the coastal ocean harbors distinct bacterial taxa implicated in CDA degradation and these taxa have not been previously identified in prior CDA degradation studies, indicating an unexplored diversity of CDA-degrading bacteria in the ocean. Moreover, the shape of the plastic article (e.g., a fabric, film, or foam) and plasticizer in the plastic matrix selected for different microbial communities. Our findings pave the way for future studies to identify the specific species and enzymes that drive CDA degradation in the marine environment, ultimately yielding a more predictive understanding of CDA biodegradation across space and time.

## INTRODUCTION

Global plastic production has increased significantly, resulting in ∼13 million tons of plastic entering the ocean annually (1–4). Such widespread plastic pollution has motivated research efforts to identify alternative plastic materials that are sustainably sourced, high functioning, and exhibit low persistence in the environment (5–7). Cellulose diacetate (CDA), a synthetic polymer made from cellulose and acetic acid, is a bio-based alternative plastic that is currently used in consumer goods, including textiles and cigarette filters, the most widely littered item on the planet (8–11).

CDA-based materials have been reported to readily degrade in a wide range of terrestrial, freshwater, wastewater, and brackish environments (12–17). Recently, we documented mass loss of CDA materials (i.e., fabric, film, and foam) in a flow-through seawater mesocosm, finding that these materials disintegrate on the timescales of months (18). The timescales of disintegration were comparable to their respiration to CO_2_, indicating that mass loss of CDA is a strong proxy for complete degradation in the coastal ocean (18). Moreover, the early onset of esterase relative to cellulase activity during the incubation, combined with measures of the stable and radiocarbon isotopic signatures of respired CO_2_ indicated that deacetylation was the rate-limiting step of CDA biodegradation in the ocean (18). However, despite broad knowledge about the lability of CDA to degradation in the environment, much less is known about microorganisms involved in the degradation, particularly in the ocean.

Previous studies have documented the biodegradation of CDA plastics in soil, landfill, and compost by several bacterial families and orders (e.g., *Neisseriaceae*, *Bacillaceae*, *Pseudomonadaceae*, and *Synergistales*) (13–15, 17). Moreover, a recent study reported that CDA was also degraded by microbes in brackish water (16), extending the potential of CDA degradation to low-salinity marine environments. However, the microorganisms implicated in the degradation of CDA materials in the ocean, especially those driving the rate-limiting deacetylation step, remain unknown.

The objective of this study was to examine the microbial community response to CDA-degradation in the coastal ocean and identify specific microorganisms that may be driving the biodegradation of diverse CDA-based materials. To achieve this objective, we incubated CDA fabric, film, and foam in a continuous-flow mesocosm with natural seawater alongside materials with high degradative capacity (cotton fabric and cellulose film) (19, 20) and low degradative capacity (polyethylene terephthalate [PET] fabric and polyethylene [PE] film) (21, 22). Mass loss, enzymatic activity, and respiration of the materials were previously reported (18). Building off of our previous findings documenting microbial degradation (18), here we report the microbial communities associated with the materials, providing an opportunity to understand how communities differ across time and material type, morphology, and formulation. Our analyses identified specific bacterial groups associated with the degradation of diverse CDA-based materials in the coastal ocean, many of which have not been associated with CDA degradation in other environments.

## RESULTS

### Overview of the experiment

Seven material types were incubated in a flow-through coastal seawater mesocosm, including CDA materials (fabric, film, and foam), positive controls (cotton fabric and cellulose film), and negative controls (PET fabric and PE film) (Fig. S1). Throughout the 13-week incubation, substantial mass loss (Fig. S2 and S3), increased activities of functional enzymes (i.e., esterases and cellulases), and stable (^13^C) and radiocarbon (^14^C) changes in mineralized CO_2_ (18) were observed for all CDA materials and positive controls. Collectively, these results indicated that microbes mediated the degradation of three morphologies of CDA materials and positive controls, but no biodegradation was observed in the negative controls. Microbial communities associated with the CDA materials and positive and negative control materials were characterized as a function of material type and incubation times, the results of which form the basis of identifying microbial communities driving CDA degradation in the seawater.

### Alpha and beta diversity

**Fig 1:**
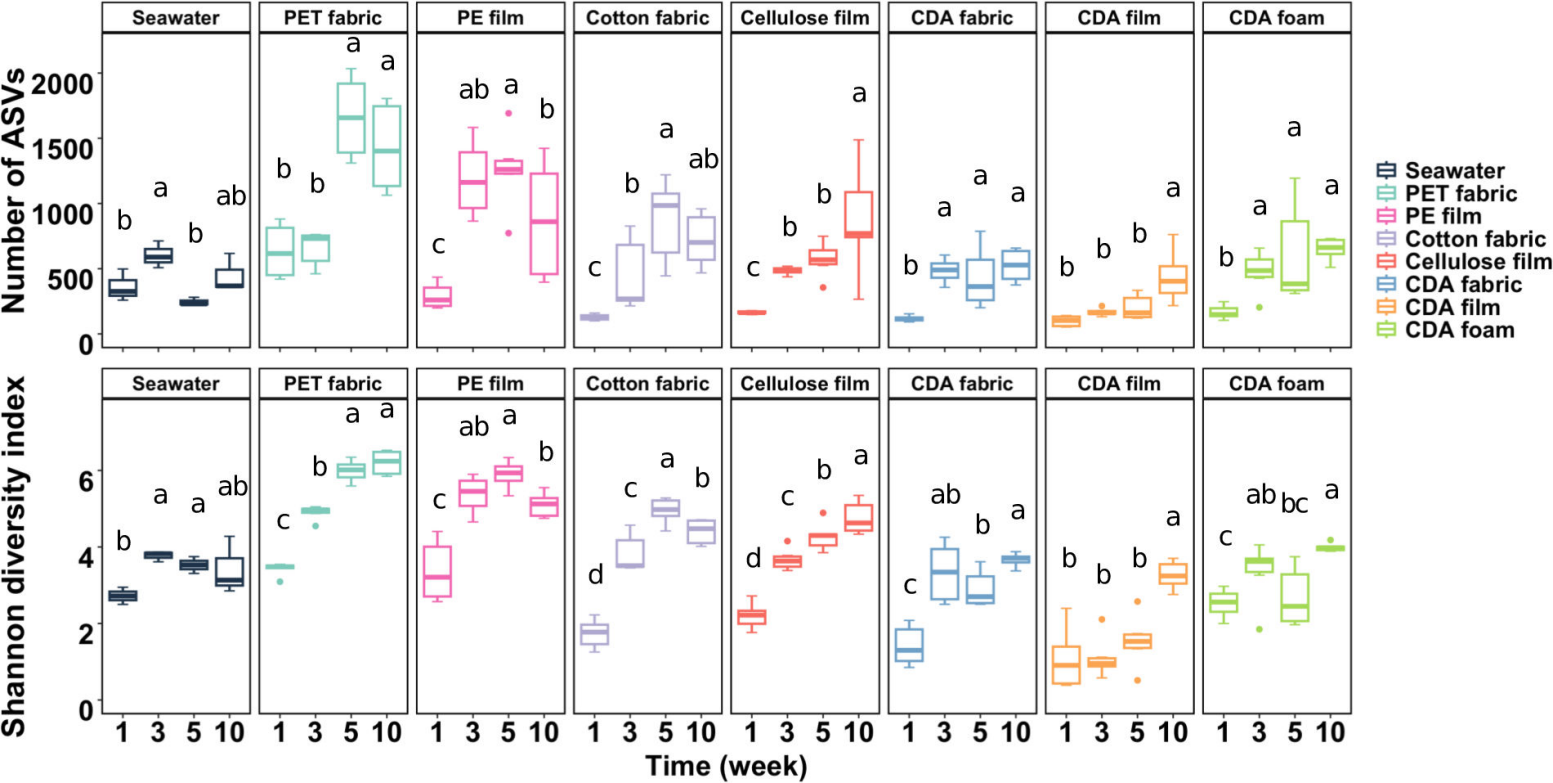
Comparison of alpha diversity of microbial communities with the number of ASVs (upper panel) and Shannon diversity index (lower panel). Different letters above boxes indicate significant differences between treatment levels within groups by comparing means using least significant difference test (*α* = 0.05). Samples with the same letter are not significantly different. Error bars represent the standard error of six datasets from two biological samples (see Materials and Methods for details).

CDA and control materials and seawater samples were collected for microbial community composition analysis at weeks 1, 3, 5, and 10 to capture microbiome signatures of initial colonization, subsequent degradation drives, and succession. Microbial communities associated with all material types and seawater were examined using SSU rRNA gene sequencing (180 samples), revealing 63,330 ± 30,387 high-quality sequences per sample and a total of 30,668 amplicon sequence variants (ASVs) in the data set (Table S1). The alpha diversity of the microbial community was significantly affected by the material type and incubation time (*P* < 0.05, Table S2). Seawater microbial community richness remained relatively stable over time (no significant difference between start and finish) (Fig. 1). Among the different material types, richness (i.e., observed ASVs) and the Shannon diversity index were highest in the two negative controls and lowest in the CDA film (Fig. 1); however, no significant differences were observed for CDA fabric and foam and two positive controls compared with the seawater. The alpha diversity for the negative controls, positive controls, and the CDA treatments increased with incubation time (Fig. 1), suggesting the reconstruction of microbial communities on the material surface.

**Fig 2:**
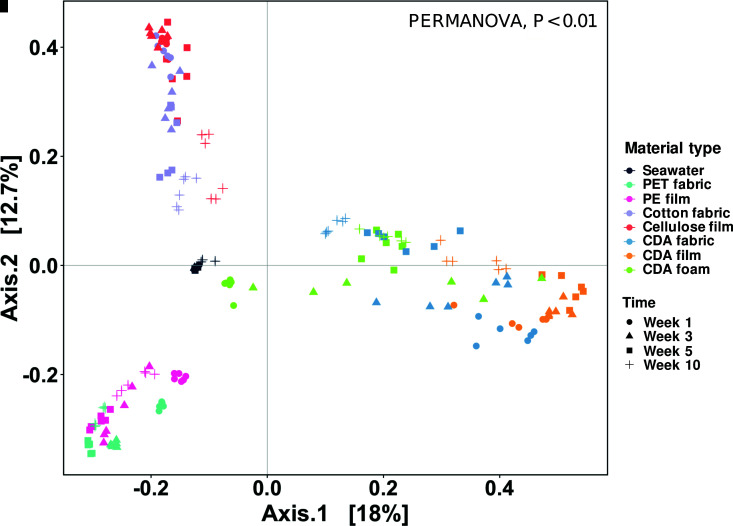
Beta diversity of microbial communities based on Bray–Curtis dissimilarity of 16S rRNA gene sequences. Samples are visualized by PCoA with colors distinguishing seawater, negative controls, positive controls, and CDA treatments. Symbol shape reflects incubation time in the flow-through seawater mesocosm.

Beta diversity analysis based on Bray–Curtis dissimilarity, a measure of the dissimilarity of different communities, indicated distinct community compositions in response to different incubation times and materials (permutational multivariate analysis of variance [PERMANOVA], *P* < 0.01) (Fig. 2; Fig. S4). The principal-coordinate analysis (PCoA) showed that ∼31% of the total variability of the microbial communities in the seawater, negative controls, positive controls, and CDA treatments at the ASV level was explained by material type and incubation time (Fig. 2; Fig. S4; Table S3). In addition, communities shifted significantly over time in negative controls, positive controls, and CDA treatments (Fig. 2; Table S3, PERMANOVA, *P* < 0.01). The communities growing on negative and positive controls reached relative stability after 5 weeks of incubation (Fig. 2; Fig. S4); however, community composition shifted markedly throughout the 10-week incubation for CDA-based materials and positive controls, suggesting that the microorganisms degrading these materials were being selected for.

In addition to incubation time, the material type was a driver of community composition. Generally, the communities growing on the same material type clustered together in the PCoA plot. That is, communities growing on CDA fabric, film, and foam all clustered together, and were distinct from the clusters of communities growing on the positive and negative controls (Fig. 2). Notably, even within the CDA clusters, distinct communities evolved between the CDA fabric, film, and foam (Fig. S5, PERMANOVA, *P* < 0.05), suggesting that material morphology and formulation (i.e., the presence or absence of a plasticizer) also drives community composition.

### Microbial community composition

The majority of the 16S rRNA gene sequences of the CDA treatments were affiliated with the phylum of Proteobacteria, with the relative abundance exceeding 95% (Fig. S6). In addition to Proteobacteria, Bacteroidota, and Myxococcota dominated in the cotton fabric and cellulose film positive controls with a combined relative abundance of up to 50% (Fig. S6). Proteobacteria dominated in negative controls of PET fabric and PE film; however, an increase in the relative abundance of Crenarchaeota and Planctomycetota was observed over a 10-week incubation period. In contrast, four major phyla, Proteobacteria, Actinobacteria, Bacteroidota, and Cyanobacteria, dominated in the seawater with a relative abundance of over 95%.

**Fig 3:**
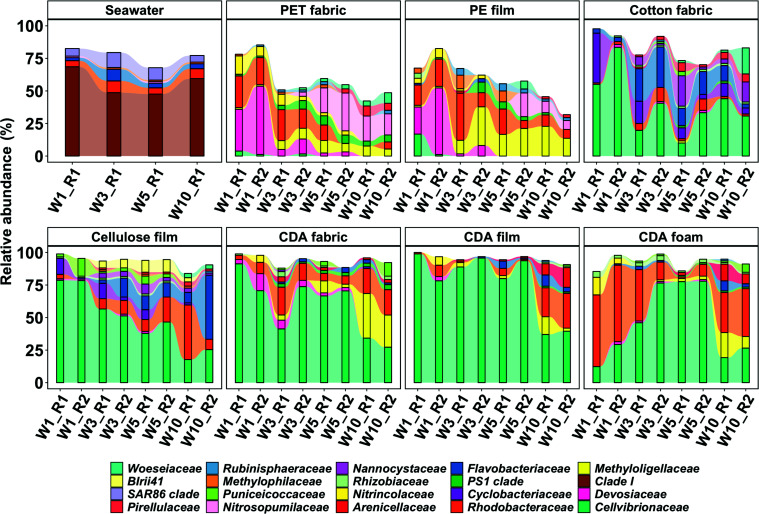
The relative abundance distributions of the top 20 ASVs at the family rank that the taxonomy was identified in the seawater, negative controls, positive controls, and CDA treatments. The numbers shown on the *x*-axis labels indicate the sampling time point (e.g., week 1 as W1) and biological replicate (e.g., replicate 1 as R1). Each biological replicate sample represents the averaged data of three datasets (see Materials and Methods Methods for details).

Further microbial community composition at the family level exhibited differences among CDA treatments, positive controls, negative controls, and seawater (Fig. 3). Two major families, *Cellvibrionaceae* and *Rhodobacteraceae*, both Proteobacteria, dominated in CDA treatments. Specifically, the relative abundance of *Cellvibrionaceae* exhibited a decreasing trend, with an initial abundance of over 80% decreasing to about 30% after 10 weeks of incubation for CDA fabric and film. Meanwhile, *Rhodobacteraceae* and *Methyloligellaceae* (both Proteobacteria) showed marked increases throughout the whole incubation period. Interestingly, the relative abundance of *Cellvibrionaceae* in the CDA foam treatment increased from ∼21% at week 1 to 78% at week 5, but reduced to 23% at week 10. In all CDA treatments, the relative abundance of *Devosiaceae* (Proteobacteria) was higher at the beginning of the incubation and lessened over time. In the positive controls, sequences of *Cellvibrionaceae* were dominant in the initial phase of the incubation but decreased to about 30% at week 10, whereas *Flavobacteriaceae* (*Bacteroidota*) and *Rhodobacteraceae* increased in relative abundance with incubation time with the combined relative abundance up to 35% in cotton fabric and 44% in cellulose film. In the negative controls, sequences representing *Devosiaceae* and *Rhodobacteraceae* exhibited a high relative abundance of 70% at week 1, while *Methyloligellaceae* and *Nitrosopumilaceae* (Crenarchaeota) became more abundant with incubation time. Notably, Alphaproteobacteria (Proteobacteria) predominated in the seawater across the whole incubation period (Fig. 3).

### Differential abundance analysis

**Fig 4:**
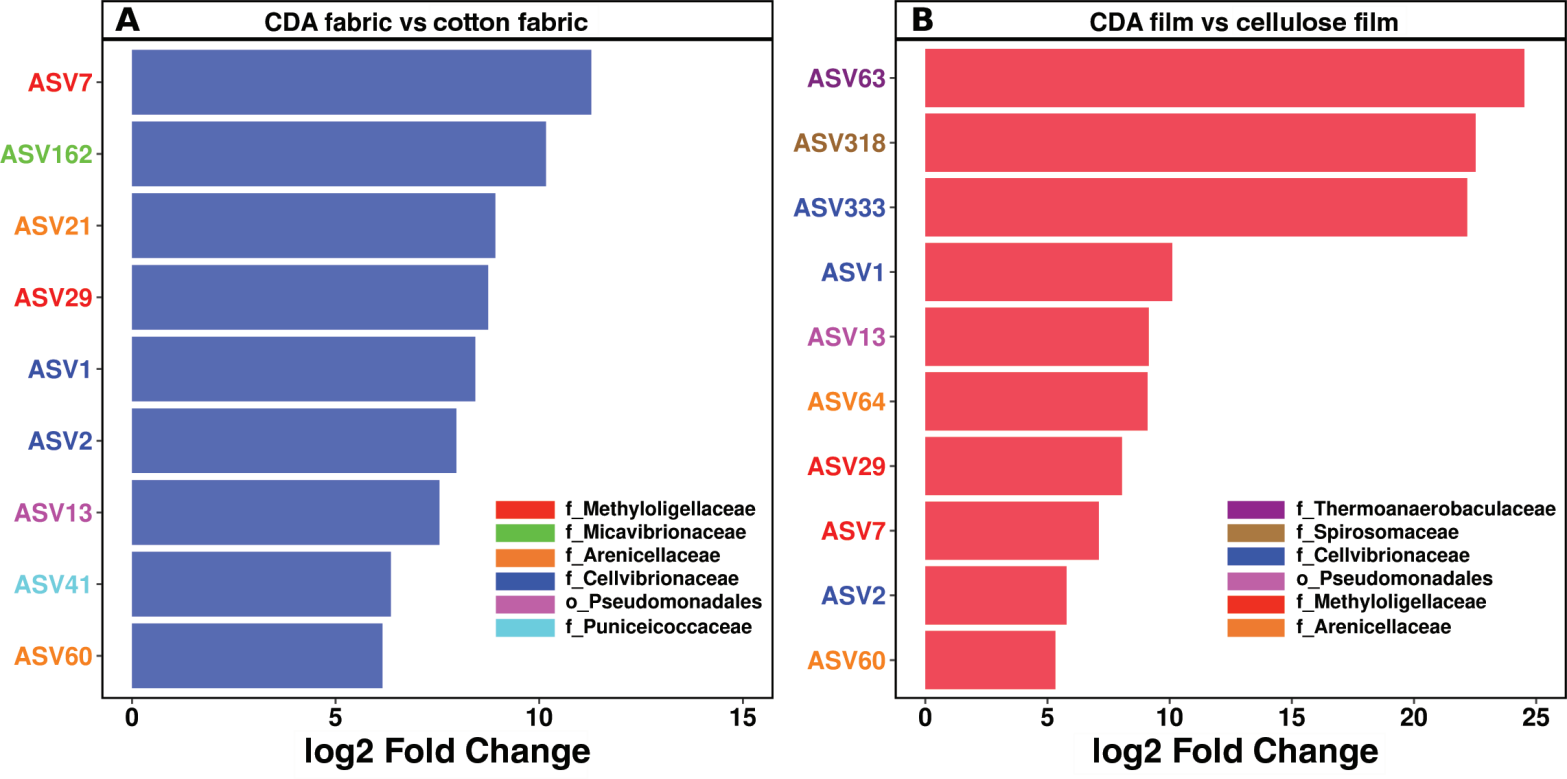
Differential abundance analysis of microbial communities (representing >0.01% of the total community) between CDA fabric and cotton fabric (**A**), and between CDA film and cellulose film (**B**), respectively. Data derived from the same type of material throughout the incubation were pooled together. The ASVs with an adjusted *P* value < 0.001 and log2 fold change >5 were considered significantly different. The colors shown on the *y*-axis labels indicate the corresponding taxonomy of ASVs with the significantly increased relative abundance of CDA fabric or film.

CDA degradation is an iterative, surface-driven process in which first the acetyl group is cleaved and then the cellulose base is degraded. By leveraging isotopic analysis and enzymatic activity assay (18), we found that the former step, deacetylation, is rate-limiting, a finding consistent with prior studies (10, 23, 24). Therefore, microorganisms that initiate deacetylation are the key drivers of CDA degradation. Differential abundance analysis of the community composition between the CDA-based material and positive control of the same morphology (i.e., fabric and film) throughout the incubation may thus reveal taxa implicated in the deacetylation of CDA (Fig. 4).

Differential abundance analysis showed nine highly responsive ASVs that markedly increased in the relative abundance of CDA fabric compared to cotton fabric, with log2 fold changes ranging from +6 to +11 (Fig. S7A). These ASVs, affiliated with the families of *Methyloligellaceae* (order_*Rhizobiales*, ASV7 and ASV29), *Micavibrionaceae* (order_*Micavibrionales*, ASV162), *Arenicellaceae* (order_*Arenicellales*, ASV21 and ASV60), and *Cellvibrionaceae* (order_*Pseudomonadales*, ASV1 and ASV2), were most significantly increased in CDA fabric (Fig. 4A). ASV1 and ASV2 exhibited the largest log2 fold changes and were both affiliated with the family of *Cellvibrionaceae*; however, the relative abundance of ASV1 decreased from about 84% to less than 1% after 10 weeks of incubation, while that of ASV2 increased from less than 1% to ∼51% (Fig. S8A). In addition, the relative abundance of ASV7 and ASV29 (family*_Methyloligellaceae*) increased significantly over time, reaching 19.2% and 7.4%, respectively, after 10 weeks of incubation.

A total of 10 ASVs were identified as markedly increased in the relative abundance of CDA film compared to that of cellulose film (Fig. 4B; Fig. S7B). These taxa were affiliated with *Thermoanaerobaculaceae* (order_*Thermoanaerobaculales*, ASV63), *Spirosomaceae* (order_*Cytophagales*, ASV318), *Cellvibrionaceae* (ASV1, ASV2, and ASV333), Pseudomonadales (ASV13), *Methyloligellaceae* (order_*Pseudomonadales*, ASV7 and ASV29), *Arenicellaceae* (order_*Arenicellales*, ASV60 and ASV64). Notably, ASV1 (*Cellvibrionaceae*) and ASV333 (*Cellvibrionaceae*) showed a marked decrease in relative abundance throughout the 10-week incubation, whereas ASV2 (*Cellvibrionaceae*) and ASV63 (*Thermoanaerobaculaceae*) exhibited the opposite trend (Fig. S8B).

## DISCUSSION

### Material degradability affects microbial community composition

Distinct microbial communities between CDA-based materials and controls were likely driven by substrate type and their surface properties. Material surface-associated microbiomes were significantly distinct from free-living communities in seawater (Fig. 2 and 3; Fig. S4), similar to what was observed in previous studies (21, 25–27); however, the communities also differed between different materials. A plausible explanation for the different community compositions between the CDA treatments, positive controls, and negative controls is the degradation of CDA samples and other cellulose-based controls (Fig. S2 and S3), which, as previously demonstrated in this exact experimental set-up (18), provided substrates for the growth of the specialized microorganisms. Meanwhile, no degradation of PET fabric and PE film were observed throughout the incubation (18), revealing that the plastics with low degradative capacity mainly provide surfaces for the colonization of microorganisms rather than substrates (27–31). Considering that the degradation of CDA bioplastics selects different microbial communities, the findings in our study have implications for elucidating the interactions between bioplastics and marine microorganisms and their potential impact on biogeochemical cycles where CDA pollution is prevalent.

The surface properties (e.g., hydrophobicity and roughness) of plastic materials could be another crucial factor for forming distinct microbial communities (31–33). For example, the hydrophobicity and hydrophilicity of plastic materials have been demonstrated to affect biofilm formation: microbes with a hydrophilic cell surface prefer hydrophilic material surfaces; those with a hydrophobic cell surface prefer hydrophobic surfaces (34, 35). PET and PE plastics are more hydrophobic than CDA materials and cellulose materials (36–40), thereby possibly selecting different microbial communities on the surface of the materials. Some dominant microbial taxa (e.g., *Devosiaceae* and *Rhodobacteraceae*) associated with PET and PE plastics in our study were also identified in previous studies conducted in the same geographic locations (41, 42). We anticipate that subtle differences between PET and PE were likely due to differences in hydrophobicity and perhaps morphology (32, 37, 38, 43), rather than their degradative capacity (18, 31).

### Plasticizer content and morphology of CDA materials affect community composition

CDA materials in the form of fabric, film, and foam, were efficiently degraded (18), but driven by different marine taxa (Fig. 3; Fig. S4), suggesting that plasticizer content and morphology exert selection on microbial communities. The CDA foam contains ∼20% (wt/wt) of the plasticizer, triacetin, which was rapidly degraded by microbes in seawater in continuous flow-through mesocosm (18). The CDA fabric and film do not contain any plasticizer (Table S4). Compared with CDA fabric and film, the rapid degradation of CDA foam (18) and distinct dominant taxa in the early phase of the incubation (Fig. 3; Fig. S5) suggests that the plasticizer is an important factor affecting both degradation rates and the microbial community composition. This finding is consistent with the similar structural features and metabolic pathways (i.e., deacetylation) between triacetin and CDA (23). Accordingly, the inclusion of triacetin may not only aid with the mechanical properties of CDA articles, but also facilitate the colonization of CDA-degrading communities and reduce environmental persistence. Future research should develop a more comprehensive understanding of the impact of plasticizers (e.g., type and content) on the biodegradation rates of CDA and other biodegradable plastics, potentially providing a design opportunity to reduce their environmental persistence.

In addition to plasticizer content, our findings demonstrate that the morphology of CDA (e.g., specific surface area) affects the colonization of microbes on the material surface. CDA fabric and film have similar chemical properties (i.e., degree of substitution, plasticizer content), yet the fabric exhibited about 1.5 times higher specific surface area than that of CDA film (Table S4). The finding that different microbial communities degrade CDA fabric and film (Fig. 3; Fig. S5) is thus consistent with expectations that specific surface area plays a critical role in shaping microbial communities on plastics (44–46). Collectively, the community variation among CDA forms likely represents the combined effects of the plasticizer content and the specific surface area.

### Unique microbial communities degrade CDA bioplastics in the coastal ocean

Our findings indicate that seawater harbors distinct microbial communities to degrade CDA bioplastics compared to terrestrial, composting, wastewater, freshwater, and brackish water environments. A number of studies have demonstrated that axenic bacteria affiliated with the families of *Neisseriaceae* (13), *Bacillaceae* (17), and *Pseudomonadaceae* (14) isolated from terrestrial and landfill environments, and taxa affiliated with the genera of *Arcobacter* and *Marinagarivorans* (16) and the family of *Synergistaceae* (15) identified in brackish water and composting environments were capable of degrading CDA bioplastics. The present study identified eight different bacterial taxa that are potentially implicated in the rate-limiting step (i.e., deacetylation) of degradation of CDA bioplastics (Fig. 4; Fig. S7; Table S5). Although *Marinagarivorans* (family_*Cellvibrionaceae*) was proposed as the major CDA degrading microbial taxa in brackish water (16), the relative abundance of this genus was negligible in our CDA samples (Fig. S6). In addition, *Marinagarivorans* was dominant in cellulose-based positive controls (Fig. S6). These findings indicate that other currently unidentified genera affiliated with *Cellvibrionaceae* were responsible for CDA degradation in coastal seawater in our study. Altogether, seven of the eight identified taxa are unique and have not been reported in prior CDA degradation studies (13–17). These unique bacterial taxa have previously been reported to colonize plastic surfaces in marine environments (27, 41, 47, 48) and some bacteria affiliated to these taxa can use a variety of organic substrates (49–51); however, this is the first linkage to the degradation of CDA bioplastics in the coastal ocean. Collectively, our findings along with those previously reported in the literature highlight that unique microbial communities in diverse environments, spanning the land to ocean continuum, readily degrade CDA bioplastics.

The discussion above provides a sensible interpretation of the 16S rRNA gene amplicon sequencing data regarding potential CDA-degrading microbial taxa; however, a few uncertainties should be considered. For example, differential abundance analysis was conducted against positive controls (i.e., cellulose) rather than negative controls (i.e., PET fabric and PE film), as has been used before. This experimental decision is justified because microbial colonization on plastic surface is controlled by their physical properties (e.g., morphology and hydrophobicity) and degradability (52–54). Considering that CDA degradation is an iterative, surface-driven process, first the acetyl group is cleaved and then the cellulose group is degraded. Therefore, the comparison between CDA bioplastic and the corresponding morphology of positive control can largely eliminate interferences caused by comparison with negative controls. In addition, the bacterial taxa potentially capable of degrading CDA bioplastics were identified by using differential abundance analysis of 16S rRNA gene amplicon sequencing data. Although this approach has been used extensively (16, 55), these findings warrant future, more in-depth investigations focusing specifically on the genetic (i.e., metagenomics) and enzymatic (i.e., metaproteomics) potential of these drivers implicated in CDA degradation in the coastal ocean.

### Environmental implications

Identifying next-generation, high-utility, and low-persistence plastic materials is a promising approach to address growing concerns of plastic contamination in the ocean and other environments. Our recent findings indicate that CDA-based materials are biodegraded to carbon dioxide in the coastal ocean on timescales of months (18). Furthermore, sunlight and photocatalytic additives, such as titanium dioxide, work synergistically to accelerate biodegradation in the coastal ocean (56). In our current study, we demonstrate that, on timescales of weeks, unique microbial communities in the coastal ocean readily colonize CDA-based materials and initiate degradation. When considering the wealth of evidence that CDA is also biodegraded to carbon dioxide in terrestrial, wastewater, and freshwater systems (12–15, 17), we propose that CDA-based materials are strong candidates for next-generation, high-utility, and low-persistence plastics. Nevertheless, it is important to stress to consumers that the identification of next-generation, high-utility, and low-persistence plastic materials does not warrant improper disposal in the environment.

While a detailed mechanistic understanding of CDA degradation has been derived from studying microbes isolated from terrestrial and wastewater environments (13, 17), we have an incomplete understanding of the physiology and ecology of microorganisms that degrade CDA in the ocean. Our findings identified several distinct taxa affiliated with families of *Arenicellaceae*, *Cellvibrionaceae*, *Methyloligellaceae*, *Micavibrionaceae*, *Puniceicoccaceae*, *Spirosomaceae*, and *Thermoanaerobaculaceae*, and the order of *Pseudomonadales* as potential targets for a more detailed exploration of the microbial species and enzymes that drive CDA degradation in the ocean. Future research should thus focus on the isolation and characterization of CDA-degrading bacteria from marine environments, potentially leading to the design of CDA bioplastics with even lower environmental persistence or novel biotechnologies for accelerated degradation of CDA waste in engineered systems.

## MATERIALS AND METHODS

### Materials

CDA fabric (97 g m^−2^), film (25 µm), and foam (510 µm) were obtained from Eastman Chemical Company (Kingsport, TN, USA). Cotton fabric (91 g m^−2^) and cellulose film (100 µm) were purchased from Huayuan Eco-Technology Company (Dezhou, SD, China) and McMaster-Carr (Elmhurst, IL, USA), respectively. PET fabric (126 g m^−2^) and PE film (25 µm) were purchased from Unifi Manufacturing Inc. (Greensboro, NC, USA). Among these seven materials, only CDA foam contains ∼20% (wt/wt) of plasticizer (i.e., triacetin) (Table S1).

### Incubation of CDA and control materials in the seawater mesocosm

Incubations of CDA and control materials were performed in a continuous flow-through seawater mesocosm with filtered natural seawater (18). The materials with low degradative capacity, PET fabric and PE film (21, 22) were used as the negative control, and the cotton fabric and cellulose film with a high degradative capacity (19, 20) were selected as the positive control. CDA materials or control materials with a size of 2.54 × 2.54 cm (length × width) were incubated in the mesocosm after sterilization with 70% ethanol and evaporation of the organic solvent. Detailed information of the mesocosm setup (Fig. S1) and seawater has been described previously (18). Seawater (salinity ~30 ppt) (57) containing native microbial communities was drawn from Martha's Vineyard Sound (Woods Hole, MA, USA), tempered to 20°C, and filtered using a 200 µm filter for removing larger particles. Filtered seawater was deposited into a head tank, providing an equal flow rate of ∼1.4 L h^−1^ with a residence time of 76 min in the mesocosm (127 × 56 × 15 cm). Experiments were performed in filtered seawater at 20°C under ambient air and light conditions. All incubations were conducted at the same time as our previous study that reported on the degradation of CDA and control materials in the flow-through mesocosm (18). An additional abiotic control experiment was conducted in 250 mL flasks with 50 mL of autoclaved seawater and demonstrated no mass loss for CDA and control materials over a month-long period (Assessed May of 2023), indicating that physical disintegration did not contribute to previous mass loss findings (18). These results are in agreement with no respiration detected in abiotic control during short-term bottle incubations (18).

### DNA extraction

For time series collection, two biological replicates per material were collected from the biodegradation experiment (see above) at week 1, week 3, week 5, and week 10, respectively. Furthermore, due to concern about the homogeneity of biomass across the materials, each replicate was snipped evenly into three parts for DNA extraction; six samples represented each time point per type of material. Collectively, 24 biofilm communities were sequenced for each material type throughout the entire experiment. Seawater samples were also taken for comparison by filtering 4 L of seawater from the mesocosm using 0.2 µm sterilized PES filters (Nalgene, Rochester, NY, USA) at identical four sampling time points. Each seawater sample contained three biological replicates. All samples were stored at −80°C prior to DNA extraction. The entire sample was subjected to DNA extraction using the DNeasy PowerBiofilm kit (Qiagen, Hilden, Germany) according to the manufacturer’s instructions, and concentrations were determined using the Qubit High Sensitivity dsDNA assay (Life Technologies, Carlsbad, CA, USA).

### 16S rRNA gene amplification and sequencing

The V4 region of the 16S rRNA gene was polymerase chain reaction (PCR) amplified from each sample using barcoded versions of the primers 515FY and 806RB (58–60). Each 50 µL PCR reaction contained 10 µL GoTaq 5× Flexi buffer, 5 µL of 25 mM MgCl_2_, 1 µL 10 mM deoxynucleotide triphosphates (dNTPs), 0.5 µL GoTaq Flexi DNA polymerase (Promega, WI, USA), 29.5 µL sterile water, 1 µL each of 10 µM forward and reverse primers, and 2 µL of extracted DNA. PCR conditions were: 2 min at 95°C, 34 cycles of 20 s at 95°C, 15 s at 55°C, and 5 min at 72°C, followed by 10 min at 72°C. Amplified products were quantity and size verified by gel electrophoresis using a 1% agarose gel, purified using the Monarch DNA Gel Extraction Kit (New England Biolabs, MA, USA) on bands excised from a 1.5% agarose gel, and quantified using the Qubit assay. Amplicons were combined in equimolar ratios and sequenced using 2 × 250 bp MiSeq platform (Illumina, Inc., San Diego, CA, USA) at the Georgia Genomics and Bioinformatics Core, University of Georgia. As a negative control for the PCRs, one reaction containing 2 µL sterile water (instead of DNA) was run with each PCR batch. No amplification was detected in any of the negative PCR controls and the one representative PCR negative control that was sequenced had a minimal number of sequences that did not pass the quality filtering and denoising steps in the bioinformatics described below. Genomic DNA from Microbial Mock Community B (HM-782D; BEI Resources, NIAID, NIH, Manassas, VA, USA) was also amplified and sequenced to assess sequencing errors.

### Bioinformatic analyses

Amplicon reads were processed in R (version 4.0.2) (61) using the DADA2 (1.16) pipeline (62) for quality control, merging sequences, and assigning ASVs. Forward and reverse reads were visually inspected for quality with DADA2 and ggplot2 (63) and to determine the cutoff values (the average number of base pairs of which quality scores fell below 30) in the filter and trim step with the following parameters: filterAndTrim(fnFs, filtFs, fnRs, filtRs, truncLen = c(240, 150), maxN = 0, maxEE = c(2), rm.phix = TRUE, compress = TRUE, multithread = TRUE). Error rates were computed and used for sequence inference in DADA2. Sequences were then merged, and ASV tables were created. Taxonomy was assigned to the family level using naïve Bayesian classifier method against the SILVA v138.1 database (64, 65), and retrieval of taxa from mock communities was checked. The taxonomy, ASV, and sample data tables were loaded into phyloseq (66), where chloroplasts and mitochondria were removed.

### Statistical analyses

Statistical analyses and plotting were performed in R. Alpha diversity (number of ASVs and Shannon diversity index) was calculated and plotted based on all ASVs counts using plot_richness included in the R package phyloseq (66). The analysis of variance (ANOVA) was performed using R package car to test the effect of material type (CDA bioplastics, controls, and seawater) and incubation time (weeks 1, 3, 5, and 10) on the alpha diversity of microbial communities (67). The significant difference between mean values of alpha diversity among the different samples within the same material was conducted using the Fisher's least significant difference test at *P* < 0.05 in R with package agricolae (68). All six replicates derived from the same sampling time point of the same material were pooled together. Beta diversity was calculated using Bray–Curtis dissimilarity and visualized using the PCoA plot in R with packages ggplot2 (63) and phyloseq (66). Statistical differences in microbial communities among different materials and incubation times were determined using PERMANOVA in R with package vegan (69).

To identify potential microbial taxa implicated in CDA degradation, differential analysis of the relative microbial abundances between CDA and corresponding positive materials was conducted using the DESeq2 (70) and the results were visualized using ggplot2 (63). As previously demonstrated (18), CDA degradation is an iterative, surface-driven process in which acetyl groups are cleaved first (i.e., deacetylation) and then cellulose groups are degraded. Therefore, the comparison between CDA bioplastic and the corresponding morphology of positive control (e.g., CDA fabric vs cotton fabric) was conducted to identify the potential microbes driving deacetylation, the rate-limiting step of CDA bioplastic degradation.

## Data Availability

The 16S rRNA gene amplicon sequencing data generated in this study were deposited in the European Nucleotide Archive under project PRJNA938436, and their respective accession numbers can be found in Table S1. Scripts for bioinformatics pipeline and statistical analyses are available upon request.

## SUPPLEMENTAL MATERIAL

Supplemental file 1 aem.01651-23-s0001.pdf Fig. S1 to S8 and Tables S1 to S4.

Supplemental file 2 aem.01651-23-s0002.xlsx Table S5.

ASM does not own the copyrights to Supplemental Material that may be linked to, or accessed through, an article. The authors have granted ASM a non-exclusive, world-wide license to publish the Supplemental Material files. Please contact the corresponding author directly for reuse.
