# Supplementary material for: Distinct microbial communities degrade cellulose diacetate bioplastics in the coastal ocean
Source: Appl Environ Microbiol. 2023 Dec 6;89(12):e01651-23. doi: 10.1128/aem.01651-23 (PMC10734458; doi:10.1128/aem.01651-23)
Supplement: Supplemental file 1 — Fig. S1 to S8 and Tables S1 to S4. [file aem.01651-23-s0001.pdf]

**Supporting Information**

# Distinct Microbial Communities Degrade Cellulose Diacetate Bioplastics in the Coastal Ocean

Yanchen Sun[†*], Michael G. Mazzotta[‡], Carolyn A Miller[†], Amy Apprill[†], Mounir Izallalen[‡], Sharmistha Mazumder[‡], Steven T. Perri[‡], Brian Edwards[‡], Christopher M. Reddy[†], and Collin P. Ward[†*]

[†]Department of Marine Chemistry and Geochemistry, Woods Hole Oceanographic Institution, Woods Hole, Massachusetts 02543, United States

[‡]Eastman Chemical Company, Kingsport, Tennessee 37662, United States

## Running title: Unique microbes degrade CDA bioplastics in the ocean

***Corresponding authors:**

Yanchen Sun, Department of Marine Chemistry and Geochemistry, Woods Hole Oceanographic Institution, 266 Woods Hole Road, Woods Hole, Massachusetts 02543, United States, Phone: +1 865-309-3246, Email: yanchen.sun@whoi.edu

Collin P. Ward, Department of Marine Chemistry and Geochemistry, Woods Hole Oceanographic Institution, 266 Woods Hole Road, Woods Hole, Massachusetts 02543, United States, Phone: +1 508-289-2931, Email: cward@whoi.edu

**Supporting information summary:**

5 tables, 8 figures and references.

**Table S1.** Information about the 16S rRNA gene amplicon data generated from the CDA degradation mesocosm.

| Time | Amplicon sequence | Seawater | Negative control | | Positive control | | CDA treatment | | |
|---|---|---|---|---|---|---|---|---|---|
| | | | PET fabric | PE film | Cotton fabric | Cellulose film | CDA fabric | CDA film | CDA foam |
| Week 1 | NCBI SRA No. | SRR23675204<br>SRR23675205<br>SRR23675206 | SRR23675226<br>SRR23675225<br>SRR23675224<br>SRR23675223<br>SRR23675222<br>SRR23675221 | SRR23675351<br>SRR23675350<br>SRR23675231<br>SRR23675220<br>SRR23675209<br>SRR23675198 | SRR23675219<br>SRR23675218<br>SRR23675217<br>SRR23675216<br>SRR23675215<br>SRR23675214 | SRR23675187<br>SRR23675260<br>SRR23675249<br>SRR23675238<br>SRR23675349<br>SRR23675338 | SRR23675213<br>SRR23675212<br>SRR23675211<br>SRR23675210<br>SRR23675208<br>SRR23675207 | SRR23675327<br>SRR23675288<br>SRR23675277<br>SRR23675266<br>SRR23675311<br>SRR23675300 | SRR23675233<br>SRR23675232<br>SRR23675230<br>SRR23675229<br>SRR23675228<br>SRR23675227 |
| Week 3 | NCBI SRA No. | SRR23675241<br>SRR23675240<br>SRR23675239 | SRR23675261<br>SRR23675259<br>SRR23675258<br>SRR23675257<br>SRR23675256<br>SRR23675255 | SRR23675203<br>SRR23675202<br>SRR23675201<br>SRR23675200<br>SRR23675199<br>SRR23675197 | SRR23675254<br>SRR23675253<br>SRR23675252<br>SRR23675251<br>SRR23675250<br>SRR23675248 | SRR23675196<br>SRR23675195<br>SRR23675194<br>SRR23675193<br>SRR23675192<br>SRR23675191 | SRR23675247<br>SRR23675246<br>SRR23675245<br>SRR23675244<br>SRR23675243<br>SRR23675242 | SRR23675190<br>SRR23675189<br>SRR23675188<br>SRR23675186<br>SRR23675185<br>SRR23675184 | SRR23675183<br>SRR23675182<br>SRR23675181<br>SRR23675264<br>SRR23675263<br>SRR23675262 |
| Week 5 | NCBI SRA No. | SRR23675285<br>SRR23675284<br>SRR23675283 | SRR23675333<br>SRR23675332<br>SRR23675331<br>SRR23675330<br>SRR23675329<br>SRR23675328 | SRR23675237<br>SRR23675180<br>SRR23675179<br>SRR23675178<br>SRR23675177<br>SRR23675176 | SRR23675326<br>SRR23675325<br>SRR23675324<br>SRR23675323<br>SRR23675322<br>SRR23675321 | SRR23675175<br>SRR23675174<br>SRR23675173<br>SRR23675172<br>SRR23675348<br>SRR23675347 | SRR23675292<br>SRR23675291<br>SRR23675290<br>SRR23675289<br>SRR23675287<br>SRR23675286 | SRR23675346<br>SRR23675345<br>SRR23675344<br>SRR23675343<br>SRR23675342<br>SRR23675341 | SRR23675340<br>SRR23675339<br>SRR23675337<br>SRR23675336<br>SRR23675335<br>SRR23675334 |
| Week 10 | NCBI SRA No. | SRR23675236<br>SRR23675235<br>SRR23675234 | SRR23675312<br>SRR23675310<br>SRR23675309<br>SRR23675308<br>SRR23675307<br>SRR23675306 | SRR23675282<br>SRR23675281<br>SRR23675280<br>SRR23675279<br>SRR23675278<br>SRR23675276 | SRR23675305<br>SRR23675304<br>SRR23675303<br>SRR23675302<br>SRR23675301<br>SRR23675299 | SRR23675275<br>SRR23675274<br>SRR23675273<br>SRR23675272<br>SRR23675271<br>SRR23675270 | SRR23675298<br>SRR23675297<br>SRR23675296<br>SRR23675295<br>SRR23675294<br>SRR23675293 | SRR23675269<br>SRR23675268<br>SRR23675267<br>SRR23675265<br>SRR23675320<br>SRR23675319 | SRR23675318<br>SRR23675317<br>SRR23675316<br>SRR23675315<br>SRR23675314<br>SRR23675313 |

**Table S2.** The ANOVA analyses for the effect of material type and incubation time on the alpha diversity of CDA materials, positive (i.e., cotton fabric and cellulose film) and negative materials (i.e., PET fabric and PE film), and seawater microbial communities. Each timepoint per type of material has six samples (see the Methods for details).

| **Alpha diversity** | **Observed ASVs** | **Shannon diversity index** |
|---|---|---|
| Material type | < 2E-16*** | < 2E-16*** |
| Time | 5.33E-13*** | < 2E-16*** |
| Material type×Time | 0.0544 | 0.0288* |

Significance level: * 0.01 < $p$-value ≤ 0.05; ** 0.001 < $p$-value ≤ 0.01; *** $p$-value ≤ 0.001.

**Table S3.** Statistical analyses of the effect of material and incubation time on microbial community composition from PERMANOVA analysis based on Bray–Curtis dissimilarity. Each timepoint per type of material has six samples (see the Methods for details).

| ***Factor*** | ***Df*** | $R^2$ | $F$ | ***p*-value** |
|---|---|---|---|---|
| Material | 7 | 0.495 | 72.498 | 0.001 |
| Time | 3 | 0.118 | 40.131 | 0.001 |
| Material×time | 21 | 0.243 | 11.829 | 0.001 |

**Table S4.** Physicochemical properties of CDA materials (1).

| | **CDA fabric** | **CDA film** | **CDA foam** |
|---|---|---|---|
| [a]Degree of substitution | 2.5 | 2.5 | 2.5 |
| Specific surface area ($m^2$ $g^{-1}$) | 0.1212 | 0.0486 | [b]NA |
| Plasticizer (Triacetin) (%, wt/wt) | 0 | 0 | 20 |

NA, not available.

[a]Degree of substitution is the average number of hydroxyl groups per repeating unit that were replaced by an acetyl group.

[b]Surface area measurements were conducted using a Micromeritics ASAP 2420 surface area analyzer (1). The measurement of the surface area of the foam was not achievable due to the leaching of the plasticizer when placed under vacuum conditions.

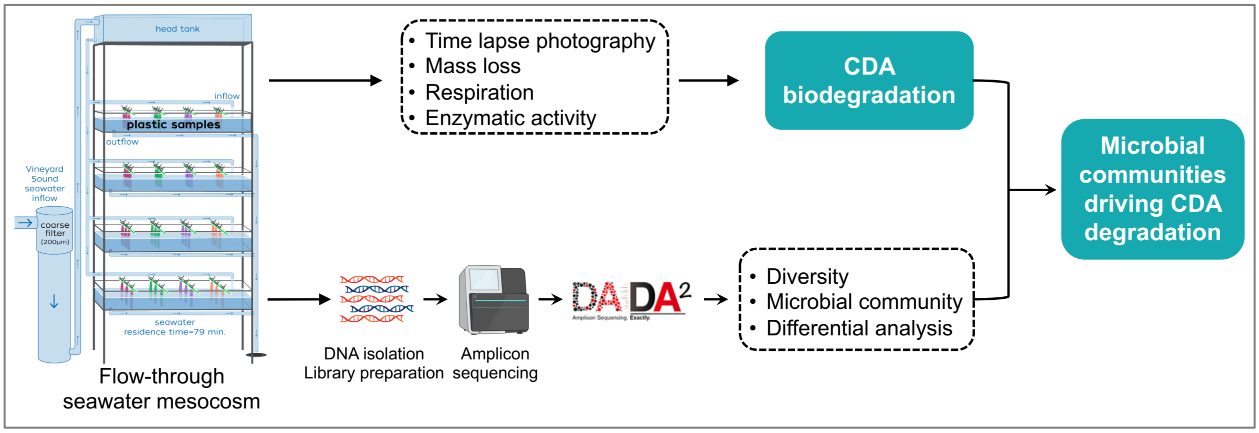


**Figure S1**. Schematic of the experimental design. Seven material types were used, including CDA materials (fabric, film, and foam), positive controls (cotton fabric and cellulose film), and negative controls (polyethylene terephthalate [PET] fabric and polyethylene [PE] film). The detailed experimental setup for the biodegradation of CDA bioplastics can also be found in our prior study (1).

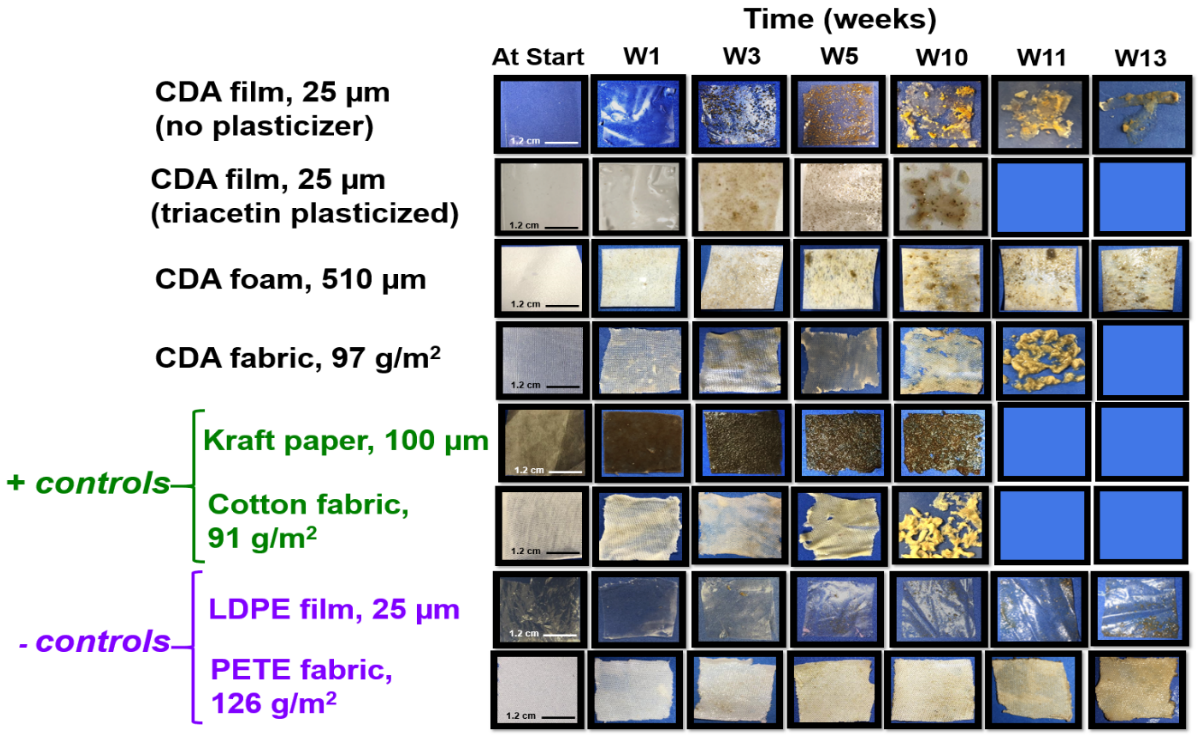


**Figure S2.** Time-lapse photography showing visual disintegration of CDA and positive controls and not negative controls over a 13-week incubation in a continuous-flow seawater mesocosm. The figure is adapted from a graph published previously (1).

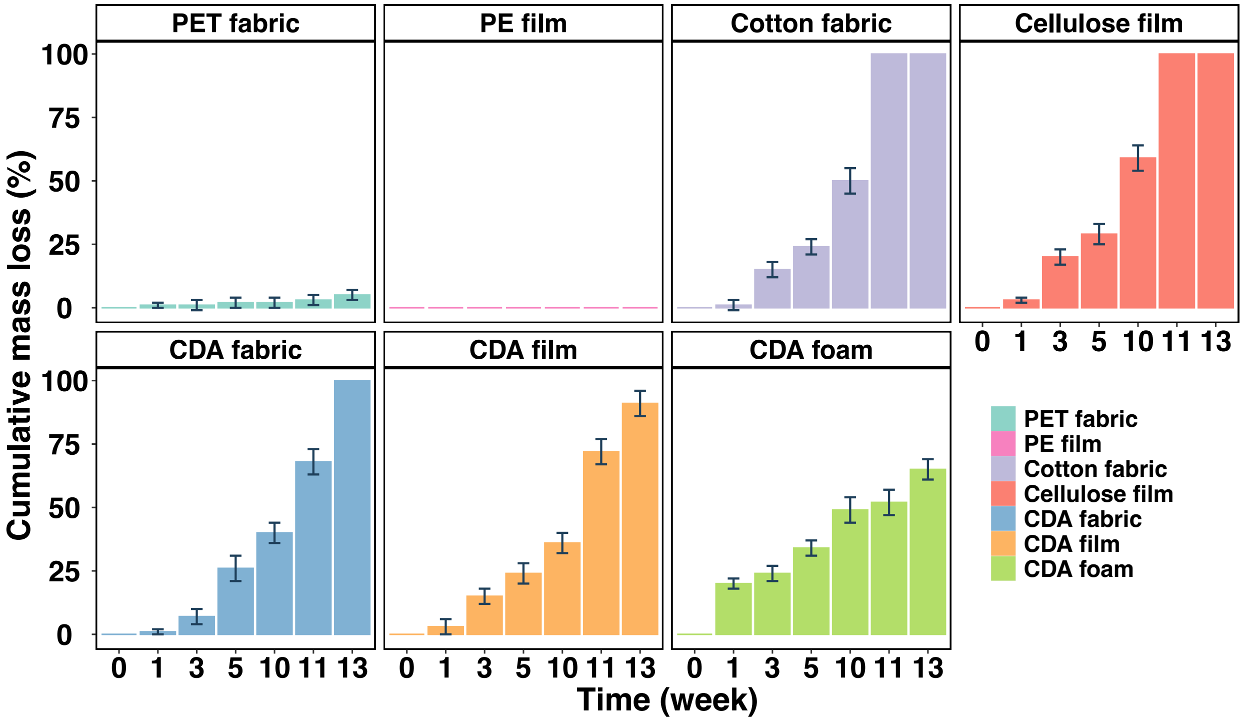


**Figure S3.** Cumulative mass loss of PET fabric and PE film (negative control), cotton fabric and cellulose film (positive control), and CDA samples (treatment) over a 13-week incubation period in a continuous-flow seawater mesocosm. Error bars represent the standard error of four replicate materials and are not depicted when no or 100% mass loss were observed. The figure is adapted from a graph published previously (1).

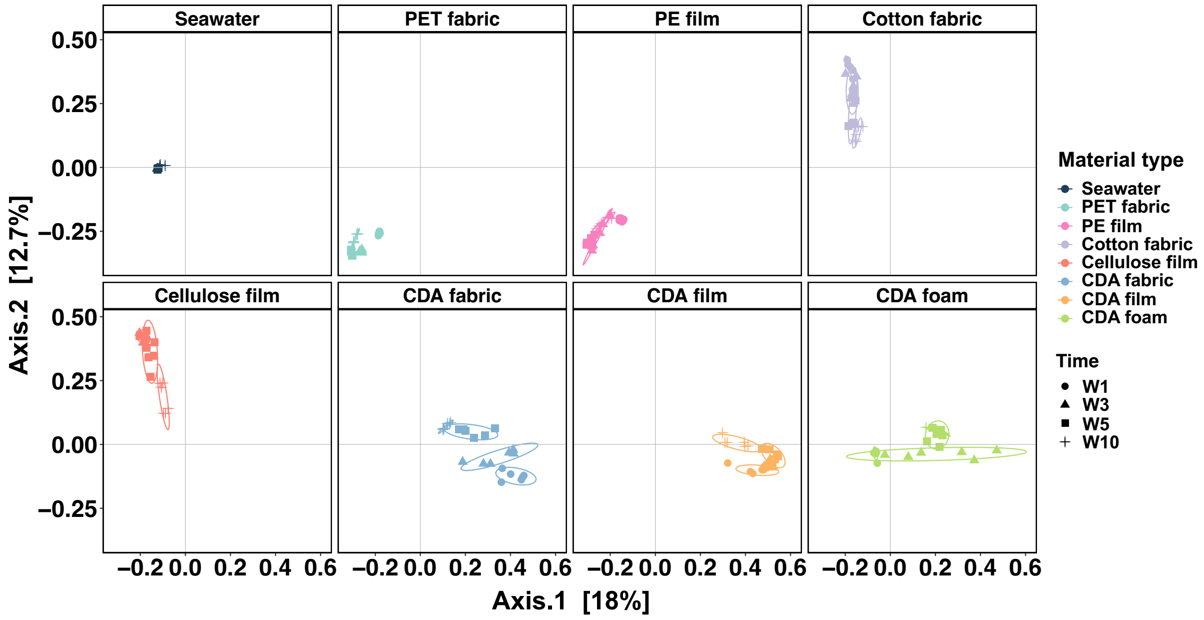


**Figure S4.** Beta diversity of microbial communities based on Bray–Curtis dissimilarity of 16S rRNA gene sequences. Samples are visualized by principal coordinates analysis (PCoA) with colors distinguishing seawater, negative controls, positive controls, and CDA treatments. The ellipses represent the 95% confidence intervals. The PCoA plot for the CDA treatment is shown in Figures S5. Microbial community composition differed significantly (PERMANOVA, $p < 0.01$) based on material type and incubation time (Table S3).

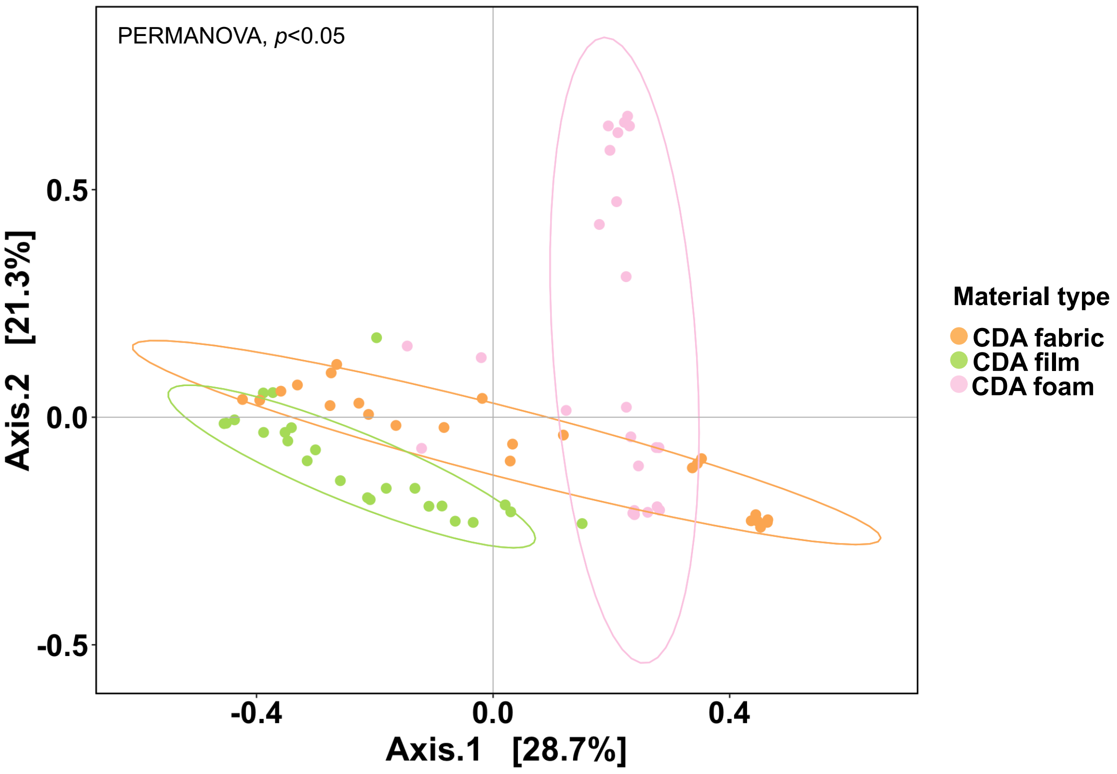


**Figure S5.** Beta diversity of microbial communities based on Bray–Curtis dissimilarity of 16S rRNA gene sequences. Samples are visualized by principal coordinates analysis (PCoA) with colors distinguishing CDA fabric, CDA film, and CDA foam. The ellipses represent the 95% confidence intervals.

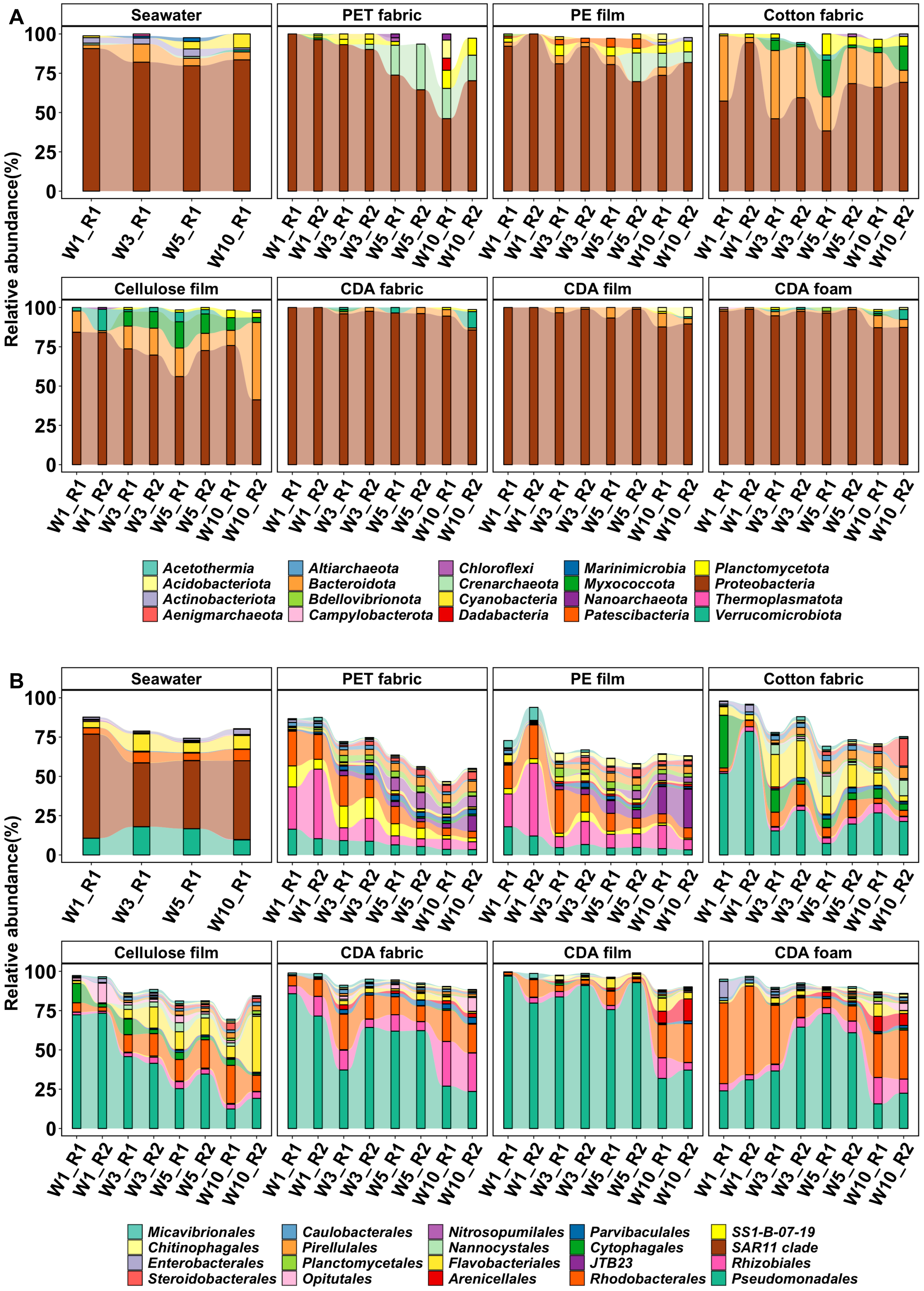
A
B
Seawater
PET fabric
PE film
Cotton fabric
Cellulose film
CDA fabric
CDA film
CDA foam
Relative abundance(%)
0
25
50
75
100
W1_R1
W1_R2
W3_R1
W3_R2
W5_R1
W5_R2
W10_R1
W10_R2
Acetothermia
Acidobacteriota
Actinobacteriota
Aenigmarchaeota
Altiarchaeota
Bacteroidota
Bdellovibrionota
Campylobacterota
Chloroflexi
Crenarchaeota
Cyanobacteria
Dadabacteria
Marinimicrobia
Myxococcota
Nanoarchaeota
Patescibacteria
Planctomycetota
Proteobacteria
Thermoplasmatota
Verrucomicrobiota
Micavibrionales
Chitinophagales
Enterobacterales
Steroidobacterales
Caulobacterales
Pirellulales
Planctomycetales
Opitutales
Nitrosopumilales
Nannocystales
Flavobacteriales
Arenicellales
Parvibaculales
Cytophagales
JTB23
Rhodobacterales
SS1-B-07-19
SAR11 clade
Rhizobiales
Pseudomonadales

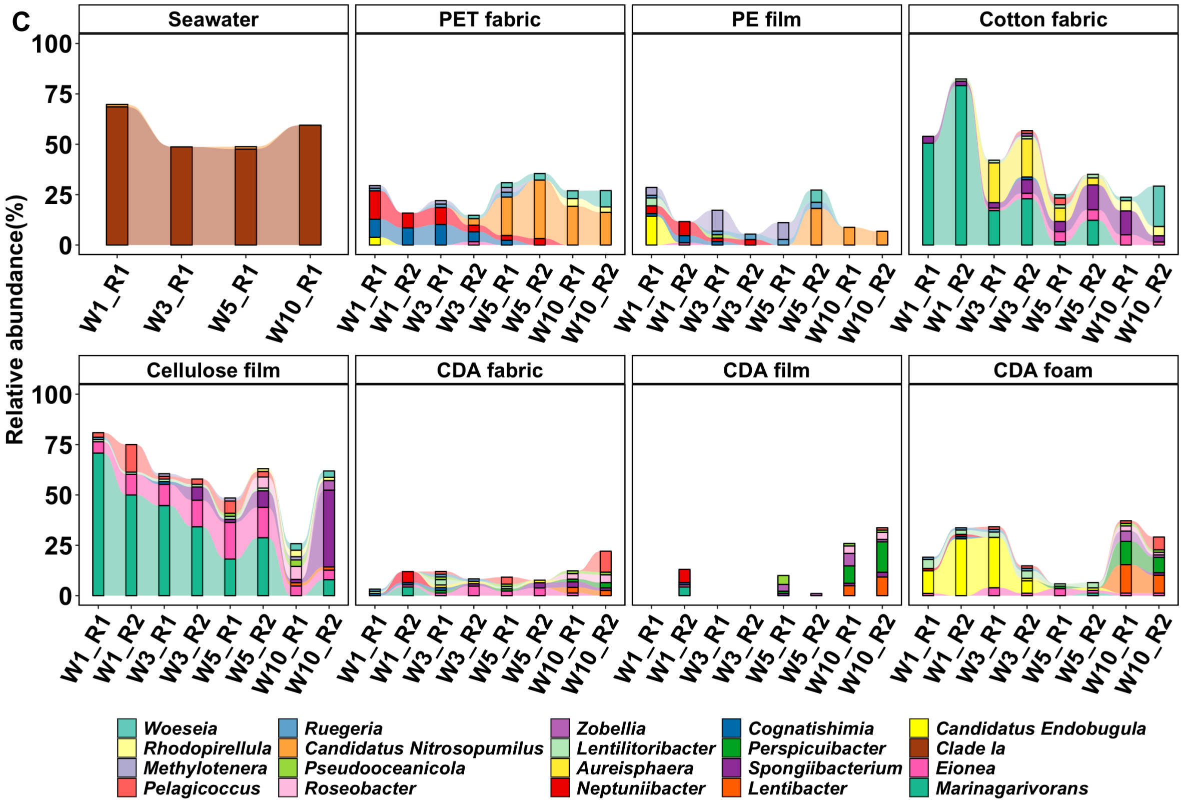


**Figure S6.** The relative abundance distributions of the top 20 phyla (A), orders (B), and genera (C) observed in the seawater, negative controls, positive controls, and CDA treatments. The numbers shown on the x-axis labels indicate the sampling time point (e.g., week 1 as W1) and biological replicate (e.g., replicate 1 as R1). Each biological replicate sample represents the averaged data of three datasets (see the Methods for details).

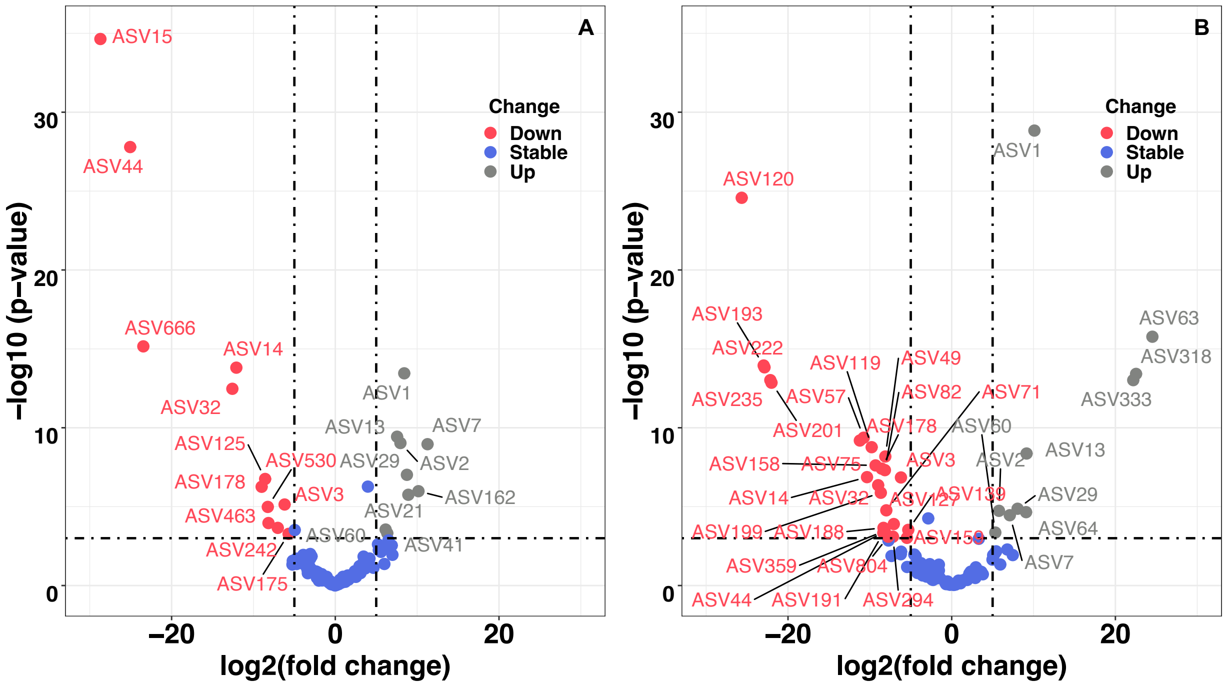


**Figure S7.** Microbial taxa (representing >0.01% of the total community) exhibited significant changes in relative abundance between CDA fabric and cotton fabric (A), and between CDA film and cellulose film (B). Data derived from the same type of material throughout the incubation were pooled together. The ASVs with an adjusted *p*-value < 0.001 and log2fold change > 5 were considered significantly different. ASVs shown in red and gray represent ASVs in CDA treatment that significantly decreased and increased in relative abundance compared to the corresponding control, respectively. ASVs shown in blue indicate no significant change in relative abundance of ASVs in CDA treatment. Taxonomy information of these ASVs can be found in the Table S5.

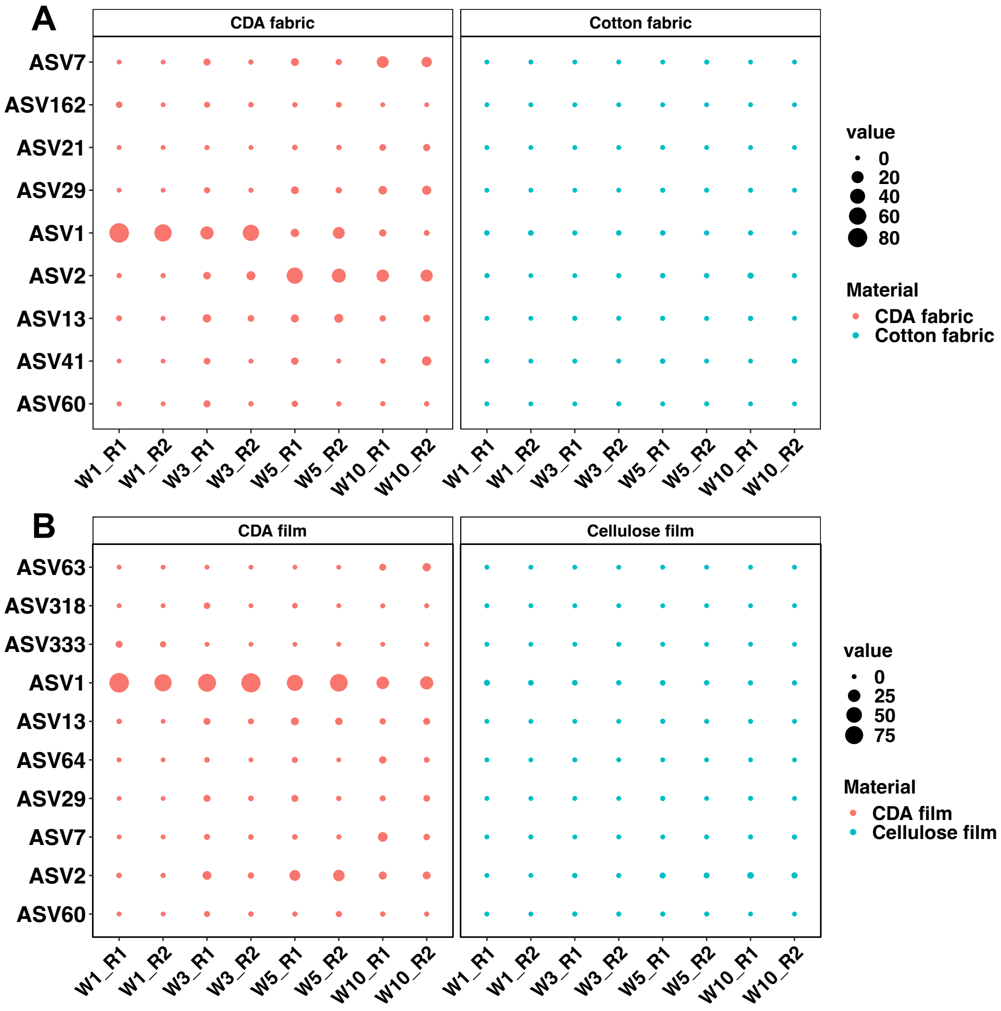


**Figure S8.** Relative abundance of significantly increased ASVs selected from CDA fabric (A) and CDA film (B). The ASVs with an adjusted *p*-value $< 0.001$ and log2fold change $> 5$ were considered significantly different. The numbers shown on the x-axis labels indicate the sampling time point (e.g., week 1 as W1) and biological replicate (e.g., replicate 1 as R1). Each biological sample replicate represents the averaged data of three datasets (see the Methods for details). Taxonomy information of these ASVs can be found in the Table S5.

**References**

1. Mazzotta MG, Reddy CM, Ward CP. 2021. Rapid degradation of cellulose diacetate by marine microbes. Environ Sci Technol Lett 9:37-41.
